# Absence of Nonlinear Coupling Between Electric Vestibular Stimulation and Evoked Forces During Standing Balance

**DOI:** 10.3389/fnhum.2021.631782

**Published:** 2021-03-25

**Authors:** Kelci B. Hannan, Makina K. Todd, Nicole J. Pearson, Patrick A. Forbes, Christopher J. Dakin

**Affiliations:** ^1^Department of Kinesiology and Health Sciences, Utah State University, Logan, UT, United States; ^2^Department of Neuroscience, Erasmus MC, University Medical Center, Rotterdam, Netherlands

**Keywords:** vestibular, coupling, galvanic vestibular stimulation, electric vestibular stimulation, random waveform, linearity, cross-frequency coupling

## Abstract

The vestibular system encodes motion and orientation of the head in space and is essential for negotiating in and interacting with the world. Recently, random waveform electric vestibular stimulation has become an increasingly common means of probing the vestibular system. However, many of the methods used to analyze the behavioral response to this type of stimulation assume a linear relationship between frequencies in the stimulus and its associated response. Here we examine this stimulus-response frequency linearity to determine the validity of this assumption. Forty-five university-aged subjects stood on a force-plate for 4 min while receiving vestibular stimulation. To determine the linearity of the stimulus-response relationship we calculated the cross-frequency power coupling between a 0 and 25 Hz bandwidth limited white noise stimulus and induced postural responses, as measured using the horizontal forces acting at the feet. Ultimately, we found that, on average, the postural response to a random stimulus is linear across stimulation frequencies. This result supports the use of analysis methods that depend on the assumption of stimulus-response frequency linearity, such as coherence and gain, which are commonly used to analyze the body’s response to random waveform electric stimuli.

## Introduction

The vestibular system encodes motion and orientation of the head in space, which is essential for navigation in, and interaction with, the world. To understand the vestibular system’s contribution to movement control, researchers use several means of probing vestibular function. One of the more common means is transcutaneous electric vestibular stimulation (EVS). EVS is the application of a small electric current to the mastoid processes behind each ear (for review: Fitzpatrick and Day, 2004). This electric current modulates the firing rate of the nearby vestibular nerves (Goldberg et al., 1982; Kim and Curthoys, 2004; Kwan et al., 2019), resulting in compensatory responses that can be recorded in the eyes (Schneider et al., 2002), ongoing skeletal muscle activity (Britton et al., 1993) and in the recipient’s posture (Wardman et al., 2003, 2004). Researchers have several options for the stimulus waveform when delivering EVS, with the most common being the step or square-wave shaped stimulus waveform often associated with galvanic vestibular stimulation. Recently, the use of random waveform electrical vestibular stimuli, rather than the traditional step or square-wave, has become more common. These stimuli have been used to induce stochastic resonance-like effects at low amplitudes (Mulavara et al., 2011), to model the postural instability that occurs following space flight (MacDougall et al., 2006; Moore et al., 2006), and for their utility as a vestibular probe (Dakin et al., 2007; Blouin et al., 2011; Reynolds, 2011; Dietrich et al., 2020; For review see Forbes et al., 2015). For this latter use, researchers often employ analysis methods that assume the stimulus’ influence on the body has a linear (one-to-one) relationship to stimulus frequencies. Until now, the linearity of the stimulus-response relationship has largely been assumed, with only cursory attention paid to this assumption (Forbes et al., 2014). However, significant non-linear behavior, such as one stimulus frequency inducing a response at a different frequency, can confound the interpretation of the body’s response to these stimuli when using analysis methods that depend on the linearity of this relationship, such as coherence and gain. Thus, the aim of this study was to examine frequency coupling linearity between an electrical stimulus and its associated postural force responses by using one of the more common stimuli, a 0–25 Hz bandwidth limited white noise, to determine the linearity of vestibular influence on posture while standing. We found that, on average, frequencies in the stimulus primarily induce responses at the same frequency in the horizontal forces acting at the feet. This result supports the use of analysis methods that operate under the assumption of frequency coupling linearity to interpret the postural response to bandwidth limited white noise EVS.

## Materials and Methods

### Participants

Forty-five participants (18 male, and 27 female, 22.07 ± 2.86 years, 172.16 ± 8.4 cm, and 70.48 ± 15.11 kg) with no known history of neurological injury or disease were included in this analysis. Each participant provided informed, written consent prior to participation. All procedures conformed to the declaration of Helsinki and were approved by Utah State University’s Institutional Review Board (protocol #9395).

### Experimental Set-Up

Upon arrival at the laboratory, participants were screened for their physical capability using a Physical Activity Readiness Questionnaire (PAR-Q) and an EVS pre-screening questionnaire that screened for potential medical contraindications to electrical stimulation near the brain. After meeting the requirements for participation, researchers placed two carbon-rubber electrodes (Covidien Uni-Patch, Dublin, IE), coated with conductive gel, bilaterally over each participant’s mastoid processes to pass the electric stimulus to the vestibular nerve. Since the direction of the postural response to EVS is dependent on head orientation, two small stickers were placed on the left side of each participant’s head to aid the monitoring of head orientation (Lund and Broberg, 1983; Fitzpatrick and Day, 2004; Khosravi-Hashemi et al., 2019). One sticker was placed at the corner of the participant’s left eye and the other sticker 18° above Reid’s plane (the line from the eye to the external auditory meatus), by the left ear, to create a level plane when the head was in the correct orientation, thus restricting the postural response to the frontal plane. The attending researcher verbally instructed the participant on how to correct their head orientation if the line made by the two stickers visually tilted from horizontal (e.g., tilt your head up slightly). During trials participants also looked at a dot on the wall in front of them to help maintain the desired head pitch.

### Procedures

**FIGURE 1:**
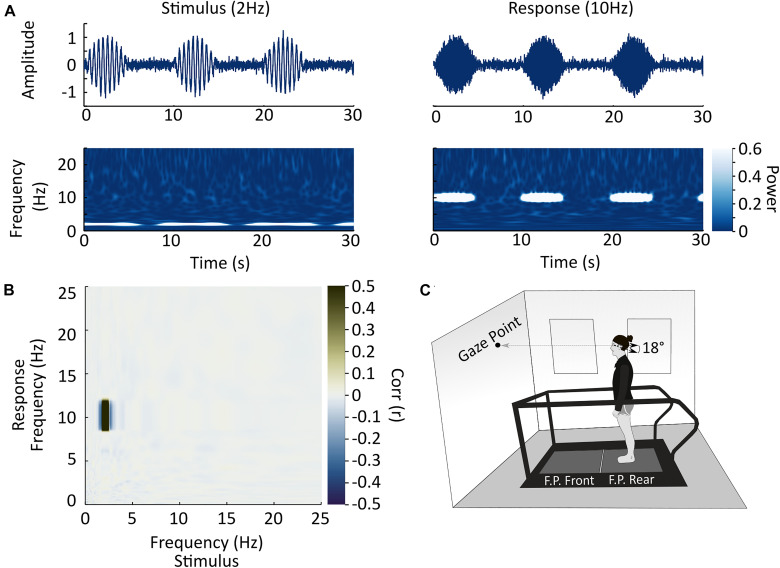
Experimental methods and setup. **(A)** Analysis example. In this study, we sought to examine co-variation in power between all stimulus and response frequencies to determine if a given frequency of stimulation resulted in a response at the same or a different frequency. The top row illustrates two simulated time-varying signals which are phase locked (co-modulated by the positive component of a 0.1 ± 0.06 Hz sinewave with negative values replaced by zeros) and vary in time at different frequencies (2 Hz stimulus inducing a 10 Hz response). The second row illustrates the modulation of signal power over time in these two time-series. Power amplitude is represented by the scale bar on the right of the figure. **(B)** In each subject, after alignment in time, we correlated the modulation in signal power over time between the stimulus and response (medio-lateral forces at the feet) for each combination of frequencies and represented these correlations as a response frequency by stimulus-frequency correlation matrix. In this simulated case, the stimulus contained 2 Hz power (see Figure 1A) and the response contained 10 Hz power, and therefore a correlation is observed at the intersection of these two frequencies. Correlation strength is represented by the scale bar on the right of the plot. **(C)** Schematic of the experimental set-up. Participants stood on the back force-plate of a two force-plate instrumented treadmill and held their gaze in an orientation meant to keep the head tilted 18° nose up. This head orientation focuses the postural response to the stimulus to the medio-lateral direction.

Prior to formal testing, participants previewed the stimulus while seated using a pair of 2-s stimulation periods with peak amplitudes of 3 and 5 mA, so they could indicate their willingness to proceed with the experiment. Once participants expressed their willingness to proceed, they stood on the back force-plate of a two force-plate instrumented treadmill (AMTI, Watertown, MA, United States) for 4 min, with their head facing forward, feet together (medial malleoli touching), and eyes open (Figure 1C). The feet were placed together to maximize the size of the stimulus-induced response (Day et al., 1997).

### Stimuli

During the 4-min standing period, participants were provided a ±5 mA bandwidth limited 0–25 Hz white noise EVS (root-mean-square of approximately 1.2 mA) created by low-pass filtering white noise using a second order Butterworth filter. Data from the force plate were also low-pass filtered at 50 Hz using a second order Butterworth filter.

### Data Analysis

To determine the coupling linearity of the stimulus-response relationship, we analyzed cross-frequency coupling [amplitude-amplitude coupling; for review see: Hyafil et al. (2015)] between stimulus and response signal power offline using custom scripts written in Julia code (Bezanson et al., 2017; Figure 1). We identified cross-frequency coupling by correlating changes in signal power over time between each stimulus frequency and medio-lateral force frequency between 0.1 and 25 Hz. To do this, we first converted the stimulus and medio-lateral force time-series to the frequency domain using a Morlet wavelet transform (Zhan et al., 2006; Blouin et al., 2011). We then circularly shifted the force signals based on the timing of the maximum cross-correlation (Rosenberg et al., 1989) between the two signals in the time-domain to align the stimulus in time with the maximum amplitude response in each subject. This process assumes a constant time lag across frequencies. The final mean shift was 322 ± 50 ms. After shifting the two signals in time, we trimmed the first and last 20 s of each signal, to remove artifacts caused by wavelet transform of the signal’s edge. We then correlated signal power over time between each frequency in the stimulus (from 0.1 to 25 Hz) and each frequency in the medio-lateral forces to provide a single pairwise Pearson correlation coefficient for each combination of frequencies in the two signals. The resulting frequency-frequency correlation matrix provides an estimate of the strength of co-variation in power between the two signals at each combination of frequencies (between 0.1 and 25 Hz).

To identify frequencies whose correlation significantly differed from zero, we performed two statistical analyses. In the first, less conservative, approach, we randomly drew, with replacement, 45 participants’ frequency-frequency correlation matrices from the empirical sample of standing trials. We calculated the mean at each frequency-frequency index across the bootstrapped sample. The bootstrapping procedure (Efron and Tibshirani, 1994) was repeated 10,000 times, after which the data was sorted to estimate the 99% confidence interval for the distribution of means at each frequency-frequency index. If the 99% confidence interval did not overlap with zero, we deemed the correlation at this frequency-frequency index to be statistically different from zero. We chose to use a 99% confidence interval because, when using data of a two-dimensional nature (i.e., varying across both time and frequency), it better represents an α-level of 0.05 (Blouin et al., 2011). In the second, more conservative, approach, we created a pseudo-sham condition by running the analysis a second time using the same stimulus signal, but inverted in time (but without shifting it in time), to de-correlate the stimulus signal from the response signal (the explanation for why this was done is below). In this second analysis, we use a similar bootstrapping procedure as used in the first analysis method. But, in addition to performing it using the stimulus-response frequency-frequency correlation matrices from each subject, with the stimulus in the correct orientation in time, we also used it on the time-inverted stimulus-response frequency-frequency correlation matrices from each subject (the pseudo-sham condition). The bootstrapped sample means for the pseudo-sham were then subtracted from the true stimulus-response means to estimate the distribution of differences in means, which was sorted, and the 99% confidence interval estimated. Frequency-frequency indices in which the 99% confidence interval for the difference in means distribution excluded zero were deemed statistically different from zero. The intuition behind this second analysis is that the pseudo-sham condition simulates the random variation in correlation around zero that would normally be present during a sham condition. However, a caveat of the pseudo-sham approach is that variation in correlation around zero may be larger than what would occur using a true sham. This is because the response signal used has greater signal power at stimulus frequencies, as it was collected during stimulation. The inclusion of uncertainty due to random variation in correlation around zero generally increases the width of the confidence intervals, decreasing discriminatory power and the risk of a type I error.

## Results

**FIGURE 2:**
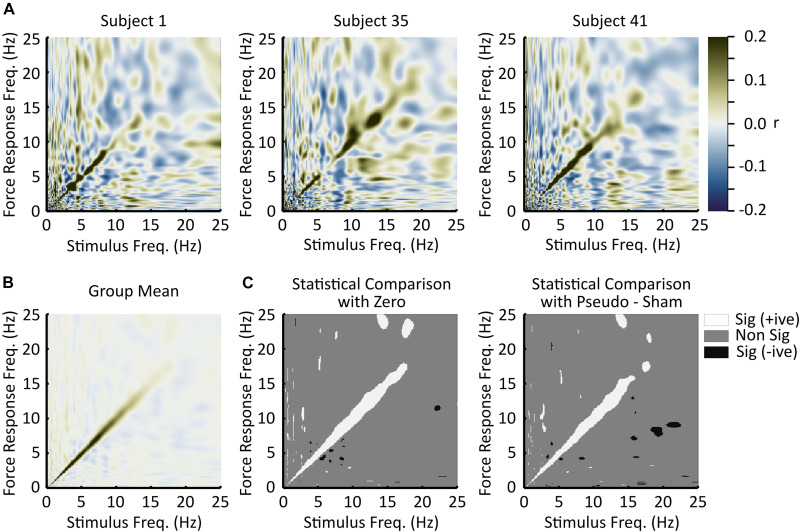
Experimental results. **(A)** Single subject responses (*n* = 1). These plots are the correlation matrices for three subjects. On the horizontal axes are stimulus frequencies and on the vertical axes are response frequencies. Correlation strength is represented by the color scale. Dark green indicates strong positive correlations, white indicates low correlations, and dark blue indicates strong negative correlations. In general, the strongest (positive) correlations were observed along the diagonal, indicating a linear frequency relationship between the stimulus and the induced responses. **(B)** Grand mean correlation matrix across all subjects. While there is high variance within each subject, on average (*n* = 45) responses induced by the stimulus were at the same frequency as the stimulus. Panel **(B)** uses the same scale bar as panel **(A)**. **(C)** Statistical evaluation. The left plot illustrates responses significantly different from zero as defined by zero being outside of the bootstrapped 99% confidence interval for the mean (*n* = 45). The right plot illustrates responses significantly different from zero defined as zero being outside of the bootstrapped 99% confidence interval of the difference of means distribution between the responses and the pseudo-sham (*n* = 45). Both statistical tests reach similar outcomes, which was that responses to the stimulus predominantly occur at the same frequency as the stimulus. In these plots, white indicates significantly greater than zero (positive correlation), gray indicates non-significance, and black indicates significantly less than zero (negative correlation).

In 42 of 45 subjects, there is a visibly discernable region of high correlation along the diagonal of the stimulus-response correlation matrix (Figure 2A and Supplementary Material). The off-diagonal responses in each subject are much more variable than the on-diagonal responses and appear to fluctuate randomly between subjects. This seemingly random off-diagonal axis behavior between subjects is largely eliminated by the averaging process, which is illustrated in Figure 2B. In both statistical tests there is a consistent region of higher positive correlation along the diagonal of the correlation matrix that is statistically different from zero (Figure 2C). Because of the dimensionality of the data, it is anticipated that small regions of significant correlations may be observed off the diagonal axis. Indeed, this is the case; however, many of these significant off-diagonal axis correlations are eliminated if the confidence interval is expanded (from 99 to 100%, using the full range of the difference distribution) to be a little more conservative, while significant correlations along the diagonal remain (Not shown).

## Discussion

Here we examined whether EVS-induced force responses recorded at the feet are linearly coupled with EVS. We observed that, on average, stimulation frequencies from 0 to 25 Hz tend to induce responses in the medio-lateral forces acting at the feet at the same frequency as the stimulus. This result provides important support for the use of measures that assume linear frequency coupling between a random-waveform vestibular stimulus and the associated medio-lateral ground reaction force response.

In recent years, it has become more common to use bandwidth limited white noise stimuli to probe the contribution of the vestibular system to motor control (Dakin et al., 2007; Mian and Day, 2009; Mackenzie and Reynolds, 2018; Tisserand et al., 2018; Dietrich et al., 2020). Often these studies assume a linear relationship between the frequencies in the input stimulus and those in the response. To date few published studies have investigated this assumption. Forbes et al. (2014) examined stimulus-response linearity using an optimized multi-sine stimulus and found that responses to the multi-sine were largely linear. Similarly, sinusoidal stimulation across a range of frequencies (1–25 Hz) results in modulation of muscle activity at the stimulus frequency with little visually observable power at other frequencies (Dakin et al., 2011). We extend these findings by demonstrating that, on average, white noise stimuli also produce force responses at the feet with a linear frequency relationship. This result is important to justify the use of analysis methods dependent upon the assumption of a linear input-output relationship. It is important to note that this relationship was present across subjects, but it is unclear whether it holds in any specific participant.

**FIGURE 3:**
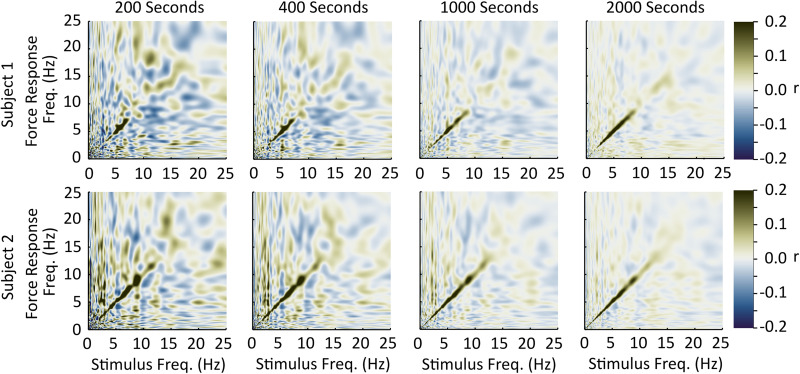
Off-diagonal axis coupling and the effect of time. We collected two additional subjects [one male, 42 years, 190 cm, 95 kg (Top row); and one female, 27 years, 163 cm, 55 kg, (Bottom row)] using the same methods as the other 45 except for the duration of stimulation. These two subjects completed two 1040-s trials to examine the influence of increasing collection duration on off-diagonal axis correlations. In these two subjects, the strength of off-diagonal axis correlations decrease with increasing collection duration, suggesting that the off-diagonal axis correlations observed in the 45 subjects are largely due to the limited collection duration.

Each subject’s correlation pattern off the diagonal axis of the stimulus-response correlation matrix was highly variable, and its spatial pattern changed between subjects. These patterns likely reflect temporary random correlations between the two signals that could be reduced through longer recording times or a stronger stimulus. To provide some support for this interpretation we collected an additional two subjects with the same methods but with an extended collection duration (Figure 3). In these two subjects, the off-diagonal axis correlations decreased with increasing collection duration, suggesting that the single subject off-diagonal axis correlations we observed are due to the 4-min recording duration. We cannot rule out, however, the possibility that some of the off-diagonal correlations could represent participant-specific non-linear coupling between stimulus and response that varies sufficiently between subjects to be averaged out over the sample. If present, these correlations appear to be small relative to the diagonal correlations.

Lastly, it is important to distinguish that, in this study, we sought to identify whether there is a linear relationship between frequencies in the vestibular stimulus and frequencies in medio-lateral ground reaction forces, as is assumed by analyses frequently applied to random-waveform EVS studies. We argue that, on average, EVS and force response exhibit linear frequency coupling over the range of stimulus amplitudes tested (0–5 mA). At first glance, these data do not appear to provide much information on other types of non-linearities that might be present in the encoding and transmission of EVS-induced responses, such as saturating non-linearities (Schneider et al., 2015). However, it is possible that such non-linearities may introduce transient non-linear coupling or frequency bleed when, for example, the stimulus amplitude exceeds a “saturating” threshold. In addition, the observation of linear coupling between stimulus and force response does not allow inference of the relationship between the stimulus and other measures such as muscle electromyography, eye motion or sway. Though, current evidence suggests that lower limb muscles may also exhibit linear frequency coupling (Dakin et al., 2011; Forbes et al., 2014).

## Conclusion

We examined the coupling linearity of the relationship between a bandwidth-limited white noise EVS and the induced behavior measured using the medio-lateral forces acting at the feet. We found that the stimulus and response frequencies had a largely linear relationship over stimulus amplitudes ranging from 0 to 5 mA, which is important to justify the appropriateness of analysis methods that depend on the linearity of the relationship.

## Data Availability Statement

The raw data supporting the conclusions of this article will be made available by the authors, without undue reservation.

## Ethics Statement

The studies involving human participants were reviewed and approved by Utah State University Institutional Review Board. The patients/participants provided their written informed consent to participate in this study.

## Author Contributions

CD and PF conceived and designed the experiments. KH, MT, and NP performed the experiments. KH and CD performed analysis of data. KH, CD, and PF performed interpretation of data. All authors contributed to the article and approved the submitted version.

## Conflict of Interest

The authors declare that the research was conducted in the absence of any commercial or financial relationships that could be construed as a potential conflict of interest.
